# Genome-Wide Association Study Reveals Novel QTLs and Candidate Genes for Grain Number in Rice

**DOI:** 10.3390/ijms232113617

**Published:** 2022-11-06

**Authors:** Peiyuan Li, Qing Li, Xueli Lu, Liping Dai, Long Yang, Yifeng Hong, Tiancai Yan, Lan Shen, Qiang Zhang, Deyong Ren, Li Zhu, Jiang Hu, Guojun Dong, Guangheng Zhang, Qian Qian, Dali Zeng

**Affiliations:** 1 State Key Laboratory of Rice Biology, China National Rice Research Institute, Hangzhou 311401, China; 2 The Key Laboratory for Quality Improvement of Agricultural Products of Zhejiang Province, Zhejiang A & F University, Hangzhou 311300, China

**Keywords:** rice, grain number per panicle, panicle branching, GWAS, haplotype

## Abstract

Grain number per panicle (GNPP), determined mainly by panicle branching, is vital for rice yield. The dissection of the genetic basis underlying GNPP could help to improve rice yield. However, genetic resources, including quantitative trait loci (QTL) or genes for breeders to enhance rice GNPP, are still limited. Here, we conducted the genome-wide association study (GWAS) on the GNPP, primary branch number (PBN), and secondary branch number (SBN) of 468 rice accessions. We detected a total of 18 QTLs, including six for GNPP, six for PBN, and six for SBN, in the whole panel and the *indica* and *japonica* subpanels of 468 accessions. More importantly, *qPSG1* was a common QTL for GNPP, PBN, and SBN and was demonstrated by chromosome segment substitution lines (CSSLs). Considering gene annotation, expression, and haplotype analysis, seven novel and strong GNPP-related candidate genes were mined from *qPSG1*. Our results provide clues to elucidate the molecular regulatory network of GNPP. The identified QTLs and candidate genes will contribute to the improvement of GNPP and rice yield via molecular marker-assisted selection (MAS) breeding and genetic engineering techniques.

## 1. Introduction

More than half of the global population chooses rice as their principal food, including over 65% of people in China [1]. In the case of limited total cultivated land, the global population is still in continuous growth, making raising grain yield per unit area one of the most important goals of rice breeding [2]. Rice yield is a complex agronomic trait composed of three elements: effective panicle number per unit area, grain number per panicle (GNPP), and grain weight [3]. The GNPP greatly varies and significantly contributes to the rice yield among the three factors [4]. Therefore, dissection of the molecular mechanisms underlying GNPP is an effective way to improve rice yield for breeders.

The rice panicle belongs to a kind of indeterminate inflorescence, which gives rise to primary and secondary branches, and spikelets on the branches. The panicle’s primary and secondary branching directly determines GNPP in rice [4]. Given the importance of GNPP in rice yield composition, researchers have performed many quantitative trait loci (QTL) mapping and identified hundreds of QTLs for GNPP-related traits. However, most QTLs were mapped in a large region (M bp), which needs further exploration. At present, several genes involved in GNPP have been isolated from QTL mappings, such as *Gnla* [5], *Ghd7* [6], *Ghd8* [7], *DEP1* [8], *IPA1/OsSPL14* [9,10], *An-1* [11], *An-2* [12], *GNP1* [13], *NOG1* [14], and *SD1* [15]. In addition, some GNPP-related genes in rice were identified through mutant-dependent map-based cloning, including *APO1* [16], *APO2* [17], *DST* [18], *GSN1* [19], *GSN4* [20], *OsSHI1* [21], *LARGE2* [22], and *OsSPL9* [23].

Among these genes, *Gnla* encodes a cytokinin oxidase and thereby degrades cytokinin. It has been identified as a major QTL contributing to rice GNPP. The decreased expression of *Gnla* leads to the accumulation of cytokinin in inflorescence meristems and increases the number of spikelets and, thus, GNPP [5]. The zinc finger transcription factor DST inhibits cytokinin accumulation in meristem by directly activating the expression of *Gn1a* and negatively regulating the number of panicle branching and GNPP [18]. Gibberellins (GAs) and cytokinin play antagonistic roles in regulating the activity of plant reproductive meristem. The higher expression of *GNP1*, encoding a GA biosynthetic protein OsGA20ox1, may improve cytokinin activity through a KNOX-mediated transcriptional feedback loop and reduce GA accumulation by increasing its catabolism activity in the rice inflorescence meristem, thus improving grain number and yield [13]. In addition, *SD1*, a paralog of *GNP1* in rice, positively regulates panicle length, branch number, and GNPP via the DELLA–KNOX pathway [15]. *Ghd7* and *Ghd8* play pleiotropic effects on heading date, panicle size, and GNPP, ultimately affecting rice yield [6,7]. *DEP1*, a major grain yield QTL, functions by determining panicle architecture [8]. The dominant allele of *DEP1* (*dep1*) is a gain-of-function mutation resulting in a truncation of the phosphatidylethanolamine-binding protein-like domain protein. The effect of the *dep1* allele is to enhance meristem activity, leading to a shortened panicle length but increased panicle branching and GNPP, as well as a consequent increase in grain yield. *IPA1/OsSPL14* acts as a critical transcription factor to control tiller outgrowth and panicle branching by directly enhancing the expression of *TB1* and *DEP1*, thereby influencing rice yield [24]. A point mutation of *IPA1/OsSPL14* disrupts the *OsmiR156*-directed degradation of *IPA1/OsSPL14*, resulting in ideal rice architecture with decreased tiller number, enhanced lodging resistance, and improved grain yield [9,10]. In contrast, OsSHI1 functions antagonistically with IPA1/OsSPL14 in regulating plant architecture by repressing the transcriptional activation activity of IPA1 towards both *TB1* and *DEP1* [21]. *An-1* and *An-2* promote awn development by enhancing rice cell division but reduce GNPP and grain yield in rice [11,12]. *APO1* encodes an F-box protein and regulates inflorescence development, thereby influencing panicle size and grain number in rice [16]. APO2 genetically and physically interacts with APO1 to cooperatively control rice panicle development [17]. In addition, *LARGE2* encodes a HECT-domain E3 ubiquitin ligase OsUPL2 and physically associates with APO1 and APO2 to modulate their protein stabilities, thereby acting with *APO1* and *APO2* in a common pathway to control panicle size and grain number [22]. *GSN1* encodes the mitogen-activated protein kinase phosphatase OsMKP1 and negatively regulates the OsMKKK10-OsMKK4-OsMPK6 signaling cascade through the dephosphorylation of OsMPK6 to determine panicle architecture [19]. In sum, the above genes generally control GNPP by affecting the differentiation of inflorescence meristem, altering panicle architecture, or pleiotropic regulating other important agronomic traits. The molecular mechanism of GNPP regulation, especially the co-regulatory network, needs further exploration.

With the advent of omics technology, the genome-wide association study (GWAS) linking genotype to phenotype has become a convenient and powerful gene/QTL mapping tool for detecting complex agronomic traits. This study aimed to apply GWAS to identify the QTLs or genes associated with GNPP in rice. We surveyed the GNPP, primary branch number (PBN), and secondary branch number (SBN) of 468 rice accessions and performed the GWAS to identify reliable loci responsible for rice panicle architecture. In total, 18 QTLs were detected, containing six for GNPP, six for PBN, and six for SBN. Among them, *qPSG1* was a common QTL for GNPP, PBN, and SBN, and was further validated using chromosome segment substitution lines (CSSLs). Combining gene annotation, gene expression, and haplotype analysis, we screened seven novel and robust candidate genes significantly associated with GNPP from *qPSG1*. The identified QTLs and candidate genes will deepen our understanding of the genetic and molecular basis for GNPP and provide essential resources for molecular rice breeding.

## 2. Results

### 2.1. Phenotypic Variation of Panicle Traits in 468 Rice Accessions

**Figure 1:**
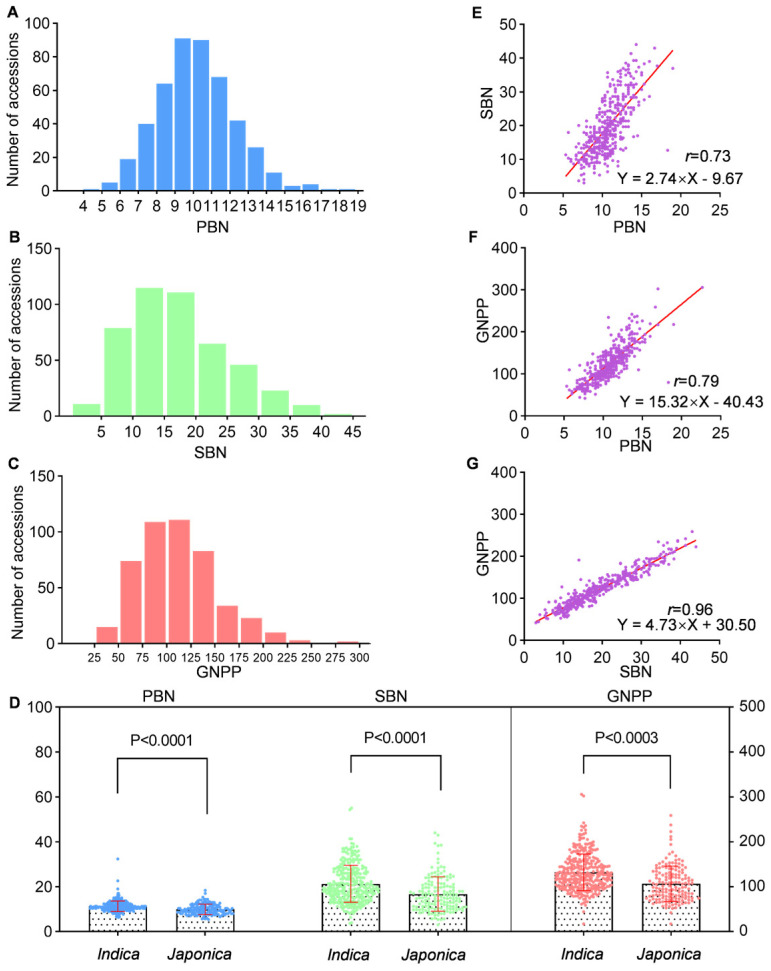
The variation of PBN, SBN, and GNPP in 468 rice accessions. (**A**) Frequency distribution of PBN. (**B**) Frequency distribution of SBN. (**C**) Frequency distribution of GNPP. (**D**) Comparison of PBN, SBN, and GNPP between *indica* and *japonica* subpanels. (**E**) One-way regression analysis between PBN and SBN among all accessions. (**F**) One-way regression analysis between PBN and GNPP among all accessions. (**G**) One-way regression analysis between GNPP and SBN among all accessions. The r values represent the Pearson correlation coefficient. The *p*-values were obtained from the *t*-test.

We surveyed three panicle traits (PBN, SBN, and GNPP) and found that they showed considerable variation in 468 accessions (Supplementary Materials Table S1). Specifically, the PBN ranged from 5.3 to 19.0, with an average of 10.7 (Figure 1A). The SBN varied from 3.0 to 55.0, with an average of 19.9 (Figure 1B). The GNPP fluctuated between 42.3 and 358.7, with an average of 125.1 (Figure 1C). In addition, all three panicle traits displayed an approximately normal distribution (Figure 1E–G). We further investigated these phenotypic differences between *indica* and *japonica* subpopulations because they are two major subspecies of Asian-cultivated rice and differ in more than 40 characteristics [25]. It showed significant differences in PBN, SBN, and GNPP between subpanels of *indica* and *japonica* (Figure 1D). The mean PBN, SBN, and GNPP in the *indica* subpanel were 11.2, 21.5, and 133.9, which are 12.96, 28.39 and 24.87% higher than those in the *japonica* subpopulation, respectively. To understand the relationship between these three traits, we performed a pairwise-correlation analysis among PBN, SBN, and GNPP using the Pearson method. The results showed strong positive correlations between PBN and SBN, GNPP, and between SBN and GNPP, with correlation coefficients (*r*) of 0.73, 0.79, and 0.96, respectively (Figure 1E–G). In conclusion, the PBN, SBN, and GNPP traits are adequate for subsequent GWAS analyses. 

### 2.2. Population Structure of 468 Rice Accessions

**Figure 2:**
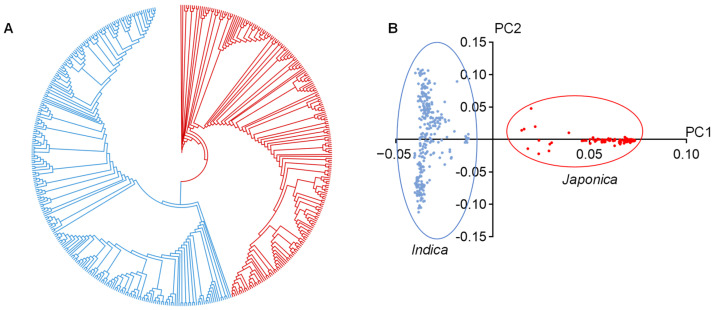
Population structure of 468 rice accessions. (**A**) Phylogenetic tree of 468 rice accessions. (**B**) Principal component analysis of 468 rice accessions. The first and second principal components are represented by PC1 and PC2, respectively. Blue and red colors correspond to *indica* and *japonica* accessions, respectively.

As shown in the phylogenetic tree, these 468 rice accessions were divided into two subpopulations close to the *indica*/*japonica* classification mentioned above (Figure 2A). Similarly, as the category of the phylogenetic tree, principal component analysis (PCA) also supported two subclades of these rice accessions (Figure 2B). In sum, these 468 rice accessions could be divided into subpanels of *indica* and *japonica*, which had similar classifications in previous studies [26].

### 2.3. Mining Loci Associated with Grain Number by GWAS

**Figure 3:**
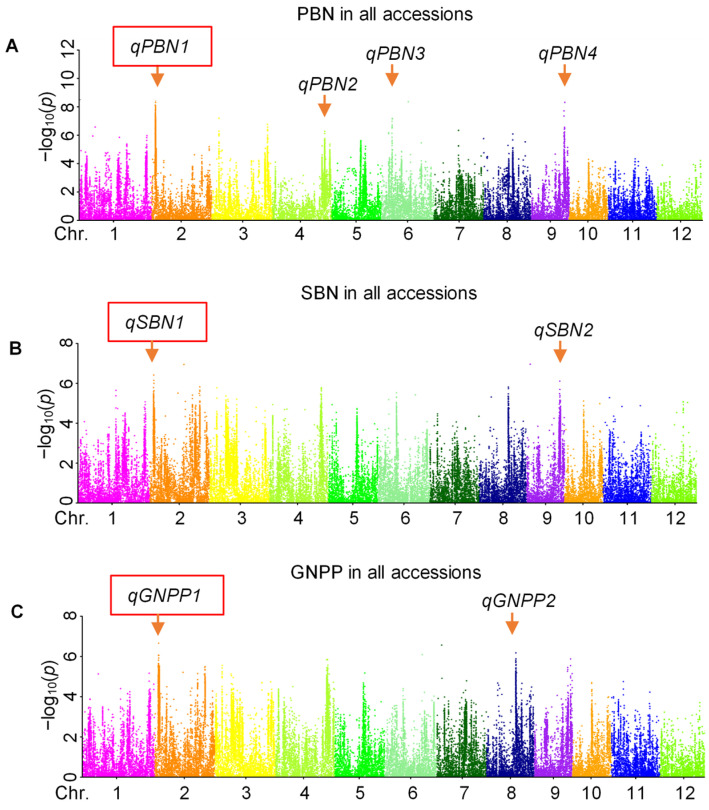
GWAS for PBN, SBN, and GNPP in 468 rice accessions. (**A**) Manhattan plot of GWAS for PBN in all accessions. (**B**) Manhattan plot of GWAS for SBN in all accessions. (**C**) Manhattan plot of GWAS for GNPP in all accessions. The red boxes indicate three overlapped QTLs.

To identify the loci affecting GNPP in rice, GWAS was carried out for the full panel of 468 accessions. As shown in Manhattan plots of GWAS, four, two, and two QTLs were detected for PBN, SBN, and GNPP, respectively (Figure 3, Table 1). Among these QTLs, *qPBN1*, *qSBN1*, and *qGNPP1* were located in the same overlapping region of chromosome (Chr.) 2 (Table 1), indicating that this region is a strong locus controlling rice grain number. Given the differences in PBN, SBN, and GNPP of *indica* and *japonica* accessions, the whole population was further divided into two subpanels for GWAS to check the effect of population structure on GWAS. The results revealed one, three, and three QTLs in the *indica* subpanel for PBN, SBN, and GNPP, respectively (Table 1, Supplementary Figure S1). However, only one QTL was found for each of the three traits in the *japonica* subpanel (Table 1, Supplementary Figure S1). In addition, these three QTLs, *qPBNj1*, *qSBNj1*, and *qGNPPj1* in the *japonica* subpanel, shared the same overlapping region on Chr. 2. Additionally, *qPBNj1* contains 154 significant single nucleotide polymorphisms (SNPs), with a lead SNP −log_10_(*p*) value as high as 10.83, which makes this overlapping region more reliable (Table 1, Supplementary Materials Table S2). In sum, a common region for PBN, SBN, and GNPP was mined from our GWAS in the whole panel and the *japonica* subpanel. The union set of six overlapped QTLs was regarded as a novel QTL controlling PBN, SBN, and GNPP simultaneously and was named *qPSG1* to simplify and avoid missing some functional regions.

### 2.4. Validation of GWAS-Associated qPSG1 Using CSSLs 

**Figure 4:**
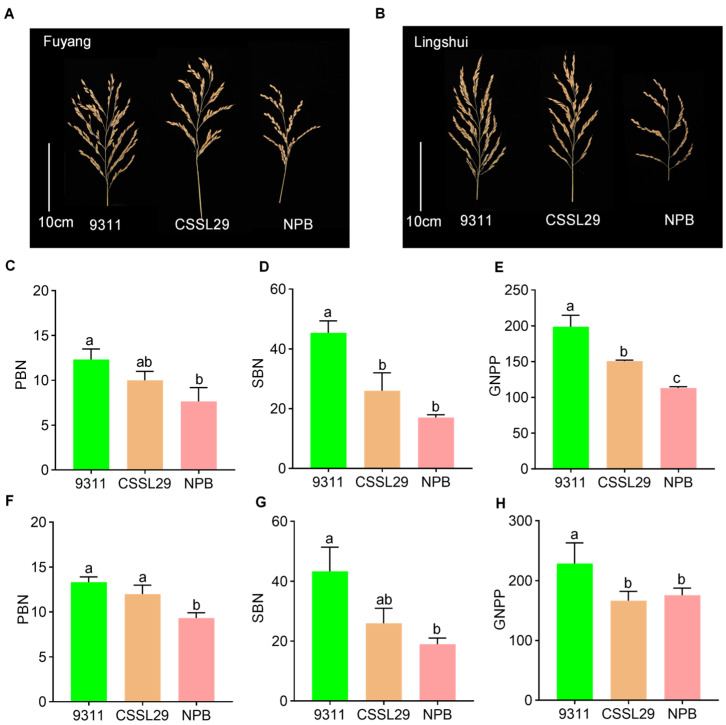
Validation of GWAS-associated *qPSG1* by CSSL. (**A**) Panicles of 9311, CSSL29, and NPB in Fuyang, Zhejiang Province. (**B**) Panicles of 9311, CSSL29, and NPB in Lingshui, Hainan Province. (**C**–**E**) Comparison of PBN (**C**), SBN (**D**), and GNPP (**E**) among CSSL29, 9311, and NPB in Fuayng, respectively. (**F**–**H**) Comparison of PBN (**F**), SBN (**G**), and GNPP (**H**) among CSSL29, 9311, and NPB in Lingshui, respectively. Data in (**C**–**H**) represent mean ± SD from ten replicates. Different lowercase letters indicate significant differences based on Duncan’s new multiple range test (*p* < 0.05).

The GWAS-associated loci were validated by screening a CSSL containing an exchanged *qPSG1* segment from the CSSL library with Nipponbare (NPB) as the donor parent and 9311 as the recurrent receptor parent and was subsequently named CSSL29. Comparing three panicle traits between 9311 and NPB, we found significant differences in PBN, SBN, and GNPP grown in both Fuyang, Zhejiang Province and Lingshui, Hainan Province (Figure 4). The 9311 panicles showed higher PBN, SBN, and GNPP than the NPB panicles. We further compared the three traits of CSSL29 and its parents and found that CSSL29 was basically in the intermediate phenotypes. In detail, compared with recurrent parent 9311, the PBN, SBN, and GNPP of CSSL29 were reduced by 18.90, 42.64, and 24.16% in Fuyang and 9.98, 40.00, and 27.11% in Lingshui, respectively (Figure 4). These data further support our GWAS results and implicate that 9311 is the dominant allele for *qPSG1*. 

### 2.5. Identification of GNPP-Related Candidate Genes in qPSG1

**Figure 5:**
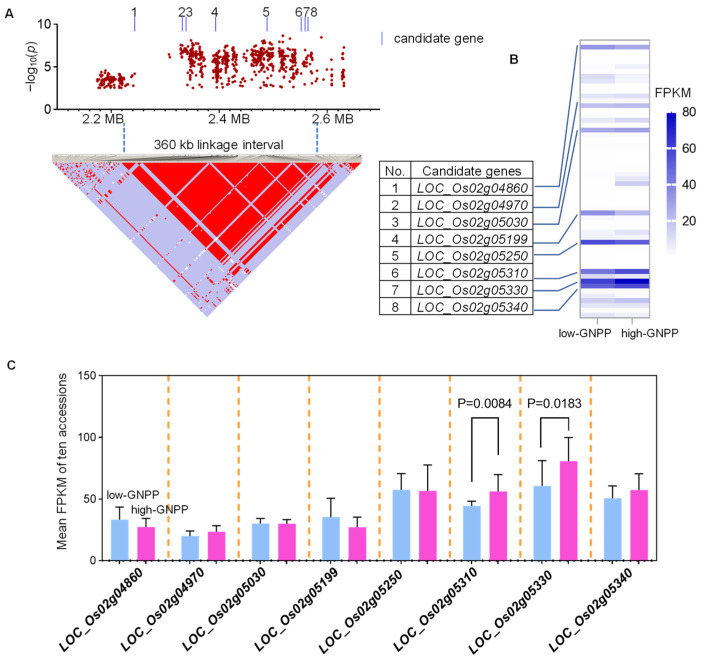
Expression levels of 57 genes in *qPSG1.* (**A**) Linkage disequilibrium plot for SNPs with −log_10_(*p*) value > 2.5 in *qPSG1* on Chr. 2. (**B**) The expression levels of all 57 genes in the 360 kb linkage interval of *qPSG1* in young panicles of ten high-GNPP and ten low-GNPP accessions from 468 rice accessions. (**C**) The expression levels of eight candidate genes in young panicles of ten high-GNPP and ten low-GNPP accessions from 468 rice accessions. Eight candidate genes with high expression are numbered from 1 to 8. The blue lines indicate the position of eight candidate genes with high expression levels. The light blue and pink bars indicate the gene expression in ten high-GNPP and ten low-GNPP accessions, respectively. Expression data represent mean FPKM values (*n* = 10). The *p*-value is obtained from the *t*-test.

**Figure 6:**
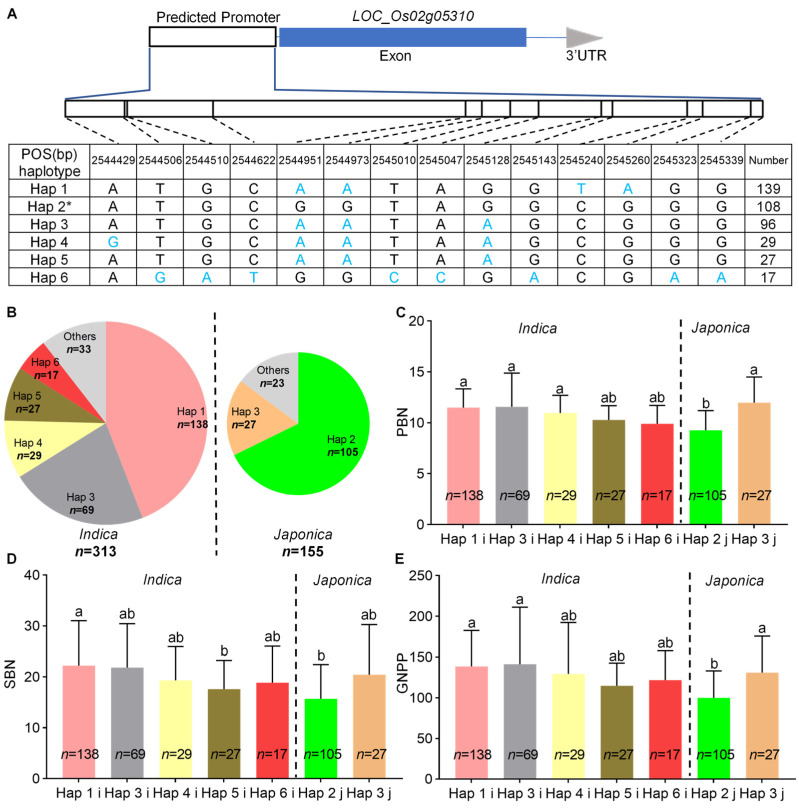
Haplotype analyses of *LOC_Os02g05310*. (**A**) Schematic representation of *LOC_Os02g05310* structure and the positions of 14 SNPs used for haplotype analysis. SNPs that differ from the reference sequence are marked with blue letters. (**B**) The haplotype frequency distribution of *LOC_Os02g05310* in the two subpanels, *indica* and *japonica*. Comparisons of (**C**) PBN, (**D**) SBN, and (**E**) GNPP among accessions with different haplotypes in the two subpanels, *indica* and *japonica*. * indicates the haplotype is the same as NPB. Others in the pie chart indicate haplotypes with a frequency of less than ten. The i and j behind Hap in (**C**–**E**) indicate *indica* and *japonica* accessions with relevant haplotypes, respectively. Different lowercase letters indicate significant differences among accessions with different haplotypes based on Duncan’s new multiple-range test (*p* < 0.05).

**Figure 7:**
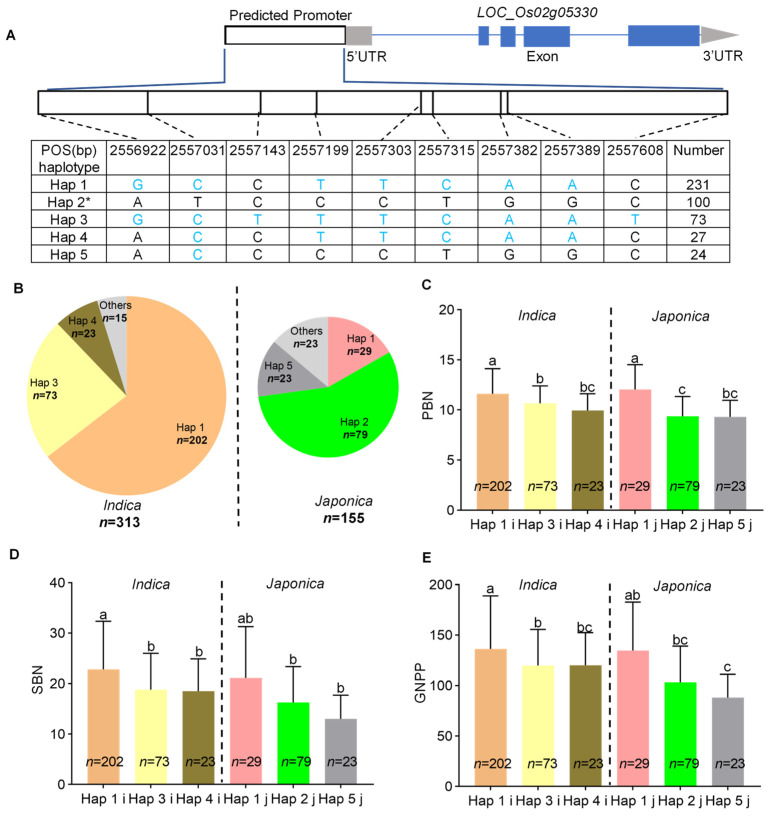
Haplotype analysis of *LOC_Os02g05330*. (**A**) Schematic representation of *LOC_Os02g05330* structure and the positions of nine SNPs used for haplotype analysis. SNPs that differ from the reference sequence are marked with blue letters. (**B**) The haplotype frequency distribution of *LOC_Os02g05330* in the two subpanels, *indica* and *japonica*. Comparisons of (**C**) PBN, (**D**) SBN, and (**E**) GNPP among accessions with different haplotypes in the two subpanels, *indica* and *japonica*. * indicates the haplotype is the same as NPB. Others in the pie chart indicate the haplotypes with a frequency of less than ten. The i and j behind Hap in (**C**–**E**) indicate *indica* and *japonica* accessions with relevant haplotypes, respectively. Different lowercase letters indicate significant differences among accessions with different haplotypes based on Duncan’s new multiple-range test (*p* < 0.05).

Based on linkage disequilibrium (LD) analysis, a 360 kb linkage interval was found in *qPSG1* (Figure 5A). The genes in this 360 kb region were further scanned using the NPB reference genome to search for known or putative genes involved in rice grain numbers. Ultimately, 57 annotated genes were identified, and no known GNPP-related gene was found in this 360 kb region. Based on the function annotations, 17 genes encoding transposon or retrotransposon protein were removed first (Supplementary Materials Table S3). In addition, ten genes with no expression in young panicles were also deleted according to our RNA-seq data (Figure 5B, Supplementary Materials Table S4). Among the remaining 30 genes, eight with high expression levels (mean FPKM > 20) in young panicles were further selected (Figure 5B). Comparing the expression levels of these eight genes in young panicles of high-GNPP and low-GNPP populations, we found that *LOC_Os02g05310* and *LOC_Os02g05330* exhibited apparent differences between the two populations from the whole panel (Figure 5C). Furthermore, *LOC_Os02g05310* and *LOC_Os02g05330* also showed noticeable expression differences between high-GNPP and low-GNPP populations from the *japonica* and *indica* subpanels, respectively (Supplementary Materials Figure S2, Table S5). We also compared the expression of these genes in young panicles of CSSL29 and 9311 and found that *LOC_ Os02g05030*, *LOC_ Os02g05199*, *LOC_ Os02g05310*, and *LOC _Os02g05330* were differentially expressed between CSSL29 and 9311 (Supplementary Materials Figure S3, Table S6). Finally, haplotype analysis was performed for eight genes using the SNPs in 468 rice accessions. To exclude the influence of the *indica* and *japonica* background, we carried out the association analysis of haplotype and phenotype in *indica* and *japonica* accessions, respectively. As a result, seven strong candidate genes with significant GNPP-related phenotypic differences between their haplotypes were further screened (Figure 6 and Figure 7, Supplementary Materials Figures S4–S8). *LOC_Os02g05250* was excluded because its three major haplotypes did not show significant GNPP-related phenotypic differences in *indica* or *japonica* accessions (Supplementary Materials Figure S9). Based on the above reasoning, we chose the two most likely candidate genes for elaboration. 

*LOC_Os02g05310* encodes the splicing factor 3B subunit 1 (SF3B1) and participates in the precursor (pre) mRNA splicing [32]. Haplotype analysis based on the SNPs in the promoter revealed that six, five, and two haplotypes existed in the whole panel, as well as the *indica* and *japonica* subpanels, respectively (Figure 6A,B). Among these six haplotypes, the haplotype (Hap) 2 accessions had less PBN, SBN, and GNPP than other Hap accessions (Figure 6C–E).

*LOC_Os02g05330*, also named *OseIF4A*, encodes a DEAD-box ATP-dependent RNA helicase. OseIF4A interacts with de novo methyltransferase (OsDRM2) at protein levels [33]. *OsDRM2* plays a crucial role in rice development, and its homozygous *osdrm2* mutants showed abnormal panicle and spikelet morphology [34]. Therefore, *LOC_Os02g05330* may control GNPP together with *OsDRM2*. Haplotype analysis depends on the SNPs in its promoter, and results showed that there are five, three, and three haplotypes in the whole panel, as well as the *indica* and *japonica* subpanels, respectively (Figure 7A,B). Among the accessions with different haplotypes, the Hap 1 accessions had the most PBN, SBN, and thus GNPP, whereas Hap 5 accessions had the least GNPP (Figure 7C–E).

## 3. Discussion

The rice panicle is relatively complex and consists of a rachis (main axis), primary branches directly attached to the rachis, secondary branches produced in primary branches, and spikelets in branches. GNPP is determined by the rate and duration of spikelet differentiation from a developmental perspective. In addition, morphologically, GNPP is directly influenced by the branch number as grains or spikelets are grown on branches [28]. Here, we investigated the PBN, SBN, and GNPP traits of 468 accessions and found a high positive correlation between them, with correlation coefficients of 0.79 between GNPP and PBN and 0.96 between GNPP and SBN (Figure 1E–G). Our study further supports that GNPP is directly affected by branching numbers, especially secondary branch numbers. The high positive correlation among PBN, SBN, and GNPP is also the reason for the similar GWAS results of these three traits, especially for SBN and GNPP (Figure 3, Supplementary Materials Figure S1). 

As one of the three factors determining rice yield, the improvement of GNPP is the main direction of rice breeding for high yield. Thus, rice research focuses on identifying the causative genes and deciphering the molecular regulatory mechanisms underlying GNPP. In the past decade, GWAS has been widely used to detect the potential loci underlying complex agronomic traits in rice. This study used three indicators, PBN, SBN, and GNPP, to perform GWAS. A total of 18 QTLs were identified through GWAS using 468 rice accessions (Figure 3, Table 1, Supplementary Materials Figure S1). Among these QTLs, at least five were co-located with previously reported GNPP-related QTLs by the traditional mapping methods, indicating that our GWAS results are quite reliable (Table 1). For instance, *qPBN1* and *qPBN2* share the same location with *Qpbn2* and *Qpbn4*, respectively, which are the two major QTLs for the primary branches per panicle [29]. *qSBNi2* corresponds to *QSbn4a*, a major QTL for the secondary branch number per panicle, and is mapped between markers RM255 and G379 [27]. *qGNPPi2* co-located with previously reported *qNOS-4-2* and *QSn4* for spikelet number per panicle [29,30]. In addition, *qGNPPi3* was included in *sn12*, a QTL reported for spikelet number per panicle [30]. When comparing our GWAS results with other GWAS results on GNPP-related traits, we found *qSBNi2* co-located with recently reported *qSBN4-3*, a QTL for the secondary branch number. *qSBN4-3* was identified for two consecutive years in the experimental fields of Sanya, Hainan Province, China, by GWAS in the *indica* subpanel [28]. However, other GWAS signals do not overlap with the previous GWAS results for GNPP-related traits [35,36,37,38,39]. In addition to the different rice accessions we used, another possible explanation for the different GWAS results is that the phenotypic statistics were derived from different geographical locations. 

We identified a common QTL, *qPSG1*, for PBN, SBN, and GNPP using GWAS in the whole panel and the *japonica* subpanel (Figure 3, Table 1, Supplementary Materials Figure S1). We further validated GWAS-associated *qPSG1* with CSSL29 (Figure 4), a relatively rare strategy in GWAS studies. Based on an LD analysis, including 57 annotated genes, a linkage interval on *qPSG1* was determined. Integrating gene annotation and expression and haplotype analysis, we screened seven strong GNPP-related candidate genes from *qPSG1*. These genes were highly expressed in young panicles (Figure 5), and some haplotypes showed differential GNPP-related phenotypes (Figure 6 and Figure 7, Supplementary Materials Figures S4–S8). Here, we selected some representative genes for discussion, except for *LOC_Os02g05310* and *LOC_Os02g05330*. 

*LOC_Os02g04970* is a homolog of *AtNup16* in *Arabidopsis*. *AtNup160* encodes a nucleoporin that prevents premature flowering by affecting the localization of HOS1 at the nuclear pore complex, which is required for the HOS1 function to degrade the CONSTANS (CO) protein [40]. Therefore, *LOC_Os02g04970* may similarly control the rice heading stage to *AtNup160*. Since there is a correlation between the heading date and panicle development, and the heading date controlling genes, *Ghd7* and *Ghd8*, also influence panicle size and GNPP in rice, *LOC_Os02g04970* may affect panicle development and GNPP by mediating the heading date. 

*LOC_Os02g05030* is annotated as a putative sucrose phosphatase, which catalyzes the final step of the sucrose biosynthesis pathway [40]. Sucrose, the primary soluble carbohydrate product of photosynthesis in higher plants, can transport long distances from source leaves to sink flowers or seeds, supporting the development of non-photosynthetic tissues. Upon entering into sink tissues, sucrose can be utilized for a wide range of cellular metabolism or converted into storage for later use [41]. Therefore, sucrose biosynthesis and allocation play pivotal roles in crops’ reproductive growth and final yield realization. Several reports have shown that improving the transport capacity of sucrose to panicles contributes to increased grain filling and rice yield [42,43]. Thus, *LOC_Os02g05030* likely regulates grain number by influencing grain filling and panicle development.

*LOC_Os02g05340* encodes a predicted RP non-ATPase (RPN) subunit of 26S proteasome, which plays a central role in the degradation of regulatory proteins involved in various developmental processes. Its homologous gene, *RPN1a*, is essential for embryogenesis and innate immunity in *Arabidopsis* [44,45]. The disruption of *RPN1a* leads to embryo lethality. Therefore, *LOC_Os02g05340* may also affect rice embryogenesis and thus control grain number.

In sum, we identified 18 QTLs for GNPP-related traits by GWAS within 468 rice accessions, especially a common *qPSG1* for GNPP, PBN, and SBN, and screened seven novel and strong candidate genes for these traits. However, to understand the natural biological function of these genes, we need to go further by overexpressing or silencing them in rice. Despite these problems, our results will lay the foundation for further study of GNPP-related traits and provide worthy genetic resources for developing high-yielding rice cultivars using genetic engineering and molecular breeding

## 4. Materials and Methods

### 4.1. Plant Materials and Phenotypes 

The 468 rice accessions, including 313 *indica* and 155 *japonica* accessions distributed worldwide with rich genetic variations, were selected for GWAS (Supplementary Materials Table S1). In November 2020, 50 net seeds were sown in the experimental field at Lingshui, Hainan, China. In April 2021, the primary tillers from at least four plants without marginal effect for each accession were harvested to determine their panicle traits, such as GNPP, PBN, and SBN. The mean data of GNPP, PBN, and SBN were presented and used for subsequent GWAS and haplotype analyses (Supplementary Materials Table S1).

Chromosome segment substitution lines (CSSLs), with 9311 as the recurrent receptor parent and NPB as the donor parent, were constructed through four times backcrosses with a molecular maker-assisted selection- (MAS) based technique [46]. CSSL29 used for *qPSG1* validation was screened from these CSSLs. The CSSL29, NPB, and 9311 were planted in Fuyang, Zhejiang Province and Lingshui, Hainan Province, China, in May 2021 and November 2021, respectively. The GNPP, PBN, and SBN data of the primary tiller for CSSL29, 9311, and NPB were measured from ten plants. 

### 4.2. SNP Genotyping

The Illumina resequencing raw data of 468 rice accessions were obtained from our lab. Read mapping and SNP calling were performed according to the previously described methods [26]. Paired-end reads for each library were mapped to the NPB reference genome IRGSP-1.0 using Burrows-Wheeler Aligner (BWA) [47]. SNP calling was conducted using the Genome Analysis Toolkit software (GATK, version 3.8.0, Cambridge, MA, USA) [48]. SNP filtering was performed using a minimum allelic frequency (MAF) greater than 5% and a missing data rate of less than 10%. Finally, a total of 3,356,591 SNPs were extracted, with an average of 8.6 SNPs per kb sequence, suggesting a high density of SNP markers in the rice genome.

### 4.3. Phylogenetic Tree Analysis

The VCF file with 3,356,591 SNP data of 468 rice accessions was used as the input file, and the relationship matrix was obtained using the software of VCF2Dis (https://github.com/BGI-shenzhen/VCF2Di (accessed on 27 September 2022)). The relationship matrix was then input into FastMe2.0 (version 2.1.6) [49] and generated a Neighbor-Joining (NJ) phylogenetic tree file. The exported phylogenetic tree was optimized by iTOL (https://itol.embl.de/ (accessed on 27 September 2022)).

### 4.4. Genome-Wide Association Study

The GWAS was conducted by the EMMAX mixed linear model method. The threshold of −log_10_(*p*) for significant SNP was set as 6.0 for SBN and GNPP but 8.0 for PBN due to the large amount of SNPs with values higher than 6.0 after considering population size and SNP number. The multi-threshold method was also used in other GWAS studies [50,51]. The Manhattan graphs were painted using the R package, Genome Association and Prediction Integrated Tool (GAPIT, http://www.maizegenetics.net/GAPIT (accessed on 27 September 2022)). Based on the previous report, 200 kb upstream and downstream of a significant SNP site in the continuous peak was considered a QTL region. The adjacent overlapped regions were regarded as the same QTL [26]. Lead SNP indicated the SNP with the highest −log_10_(*p*) value in a QTL region.

### 4.5. Linkage Disequilibrium (LD) Analysis

Pairwise linkage disequilibrium (LD) among all accessions was based on filtered SNPs with −log_10_(*p*) value > 2.5 in *qPSG1*, and then Haploview (version 4.2) was used to carry out the LD analysis (http://www.broad.mit.edu/mpg/haploview/ (accessed on 27 September 2022)).

### 4.6. RNA-Seq Analysis

Ten rice accessions with the lowest and highest GNPP were selected from the whole panel and the *indica* and *japonica* subpanels (Supplementary Materials Tables S4 and S5). Their total RNA was extracted from the young panicles with 0–1 cm length using the Trizol reagent (Invitrogen). Similarly, the total RNA of CSSL29 and its recurrent parent, 9311, were also extracted from the young panicles. The Illumina libraries were constructed following the manufacturer’s instructions (Illumina, San Diego, CA, USA). High-throughput RNA sequencing was conducted using the Illumina NovaSeq 6000 platform. Clean reads were aligned to the rice NPB reference genome (IRGSP-1.0) with HISAT2 (version 2.0.1) [52]. Quantitative expression analysis of each gene was performed with HTSeq (version 0.6.1) [53]. The mean fragments per kilobase of exon per million mapped fragments (FPKM) value of three biological replicates was regarded as the gene expression value.

### 4.7. Haplotype Analysis

Haplotype analysis for each gene in 468 accessions was preferentially performed using nonsynonymous SNPs in CDS. If no nonsynonymous SNP existed in CDS, the SNPs located in the hypothetical promoter (2 kb upstream of 5’UTR) were used for haplotype analysis. The haplotypes with a frequency of more than ten were used for further phenotypic association analyses.

## Figures and Tables

**Table 1:** A total of 18 GWAS regions are associated with the number of rice grains.

Detected Trait	Group	QTL	Chr.	Physical Region (nt)	Significant SNPs	Lead SNP		Co-Location Loci (Reference)
						Position (nt)	−log_10_(*p*)	
PBN	all	*qPBN1* *	2	2,247,586–2,689,197	7	2,517,880	8.64	*QPbn2* [27]
	all	*qPBN2*	4	25,922,967–26,322,167	1	26,122,967	8.36	*QPbn4* [27]
	all	*qPBN3*	6	15,750,260–16,150,660	1	15,950,460	8.36	
	all	*qPBN4*	9	19,819,916–20,288,743	4	20,019,916	10.69	
	*indica*	*qPBNi1*	11	24,250,379–24,650,379	3	24,450,379	8.42	
	*japonica*	*qPBNj1* *	2	2,150,634–2,630,974	154	2,350,634	10.83	
SBN	all	*qSBN1* *	2	2,330,919–2,730,919	2	2,530,919	6.15	
	all	*qSBN2*	9	19,942,726–20,342,726	1	20,142,726	6.11	
	*indica*	*qSBNi1*	2	25,653,167–26,053,167	14	25,853,167	6.15	
	*indica*	*qSBNi2*	4	29,672,943–31,234,590	4	30,724,432	6.44	*QSbn4a* [27], *qSBN4-3* [28]
	*indica*	*qSBNi3*	12	18,302,808–18,940,801	21	18,449,870	8.18	
	*japonica*	*qSBNj1* *	2	2,218,262–2,630,654	2	2,218,262	6.01	
GNPP	all	*qGNPP1* *	2	2,283,931–2,683,931	2	2,483,931	6.66	
	all	*qGNPP2*	8	17,369,016–17,769,016	1	17,569,016	6.18	
	*indica*	*qGNPPi1*	2	25,554,962–25,954,962	18	25,754,962	6.55	
	*indica*	*qGNPPi2*	4	29,672,943–31,261,400	65	31,235,111	7.38	*qNOS-4-2* [29], *QSn4* [30]
	*indica*	*qGNPPi3*	12	18,161,765–18,940,801	9	18,450,273	7.62	*sn12* [31]
	*japonica*	*qGNPPj1* *	2	2,180,189–2,637,259	12	2,193,580	6.44	

Note: * indicates the six overlapped QTLs.

## Data Availability

The data supporting the findings of this study are available in the article and its supplementary materials. The raw sequencing data presented in this study are available from the corresponding authors upon reasonable request.

## Supplementary Materials

The following supporting information can be downloaded at: https://www.mdpi.com/article/10.3390/ijms232113617/s1.
