# Supplementary material for: Genome-Wide Association Study Reveals Novel QTLs and Candidate Genes for Grain Number in Rice
Source: Int J Mol Sci. 2022 Nov 6;23(21):13617. doi: 10.3390/ijms232113617 (PMC9658369; doi:10.3390/ijms232113617)
Supplement: Supplementary file 1 [file ijms-23-13617-s001.zip › Supplementary Figures S1-S9.pdf]

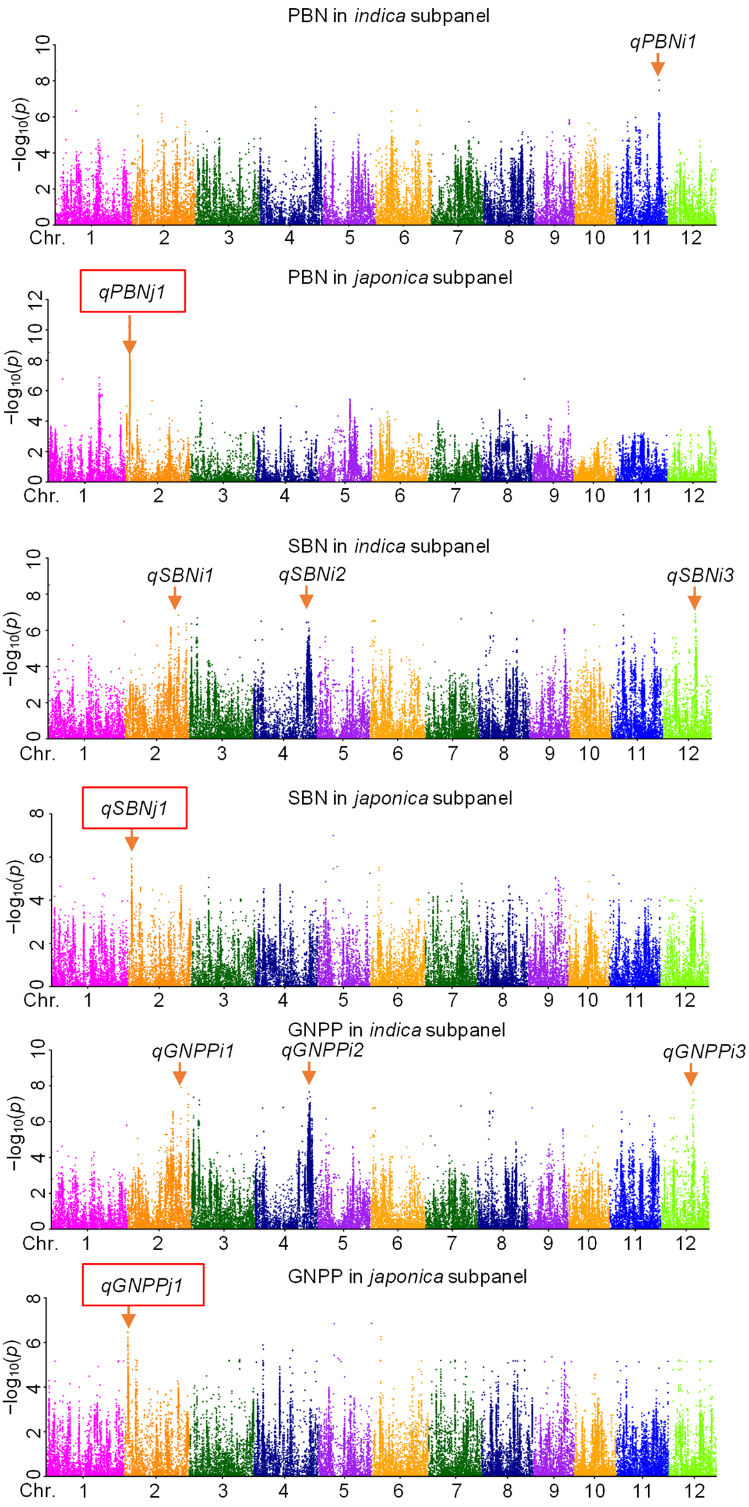

Figure S1. GWAS for PBN, SBN, and GNPP in *indica* subpanel and *japonica* subpanel. Manhattan plots of GWAS for PBN (A), SBN (C), and GNPP (E) in *indica* subpanel. Manhattan plots of GWAS for PBN (B), SBN (D), and GNPP (F) in *japonica* subpanel. The red boxes indicate three overlapped QTLs.

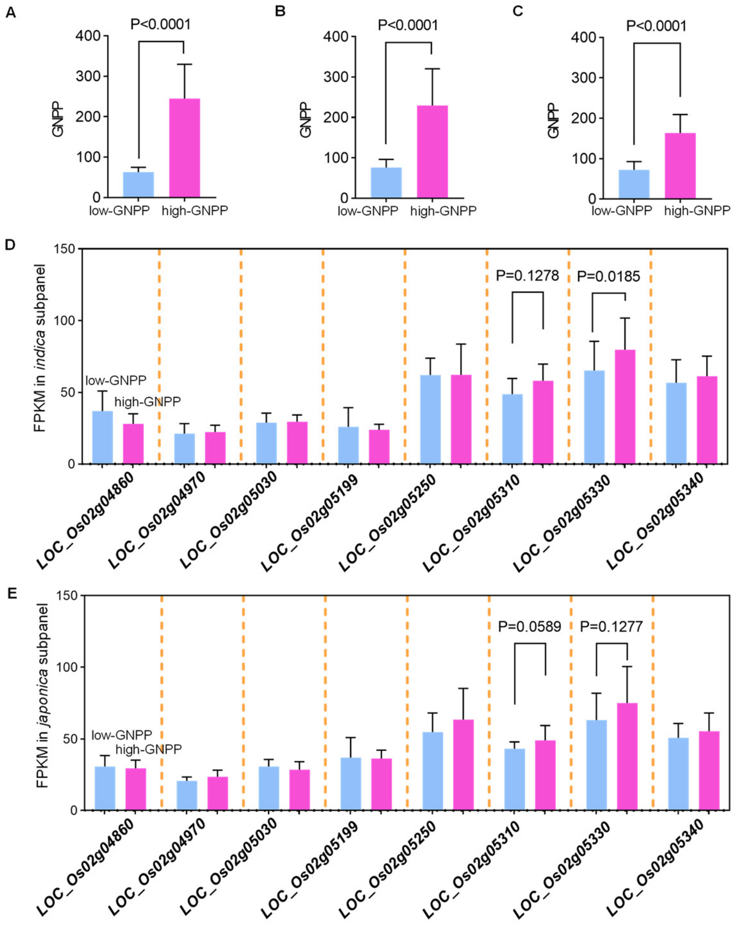

Figure S2. The mean expression levels of eight candidate genes in young panicles of *indica* accessions and *japonica* accessions. (A) Comparison of GNPP between ten high-GNPP and ten low-GNPP accessions from 468 rice accessions. (B) Comparison of GNPP between ten high-GNPP and ten low-GNPP accessions from *indica* subpanel. (C) Comparison of GNPP between ten high-GNPP and ten low-GNPP accessions from *japonica* subpanel. (D) The expression levels of eight candidate genes in young panicles of ten high-GNPP and ten low-GNPP accessions from *indica* subpanel. (E) The expression levels of eight candidate genes in young panicles of ten high-GNPP and ten low-GNPP accessions from *japonica* subpanel. Data represent mean ± SD (n=10). The p-value is obtained from the T-test.

No.	Candidate genes	P value for t-test
1	*LOC_Os02g04860*	>1
2	*LOC_Os02g04970*	0.9998
3	*LOC_Os02g05030*	<0.0001
4	*LOC_Os02g05199*	<0.0001
5	*LOC_Os02g05250*	0.1030
6	*LOC_Os02g05310*	0.0004
7	*LOC_Os02g05330*	<0.0001
8	*LOC_Os02g05340*	>1

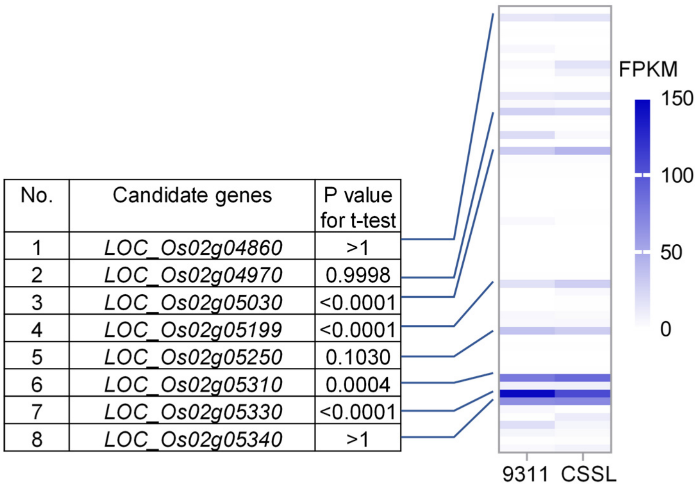

Figure S3. Comparison of the expression levels of 57 genes in *qPSG1* between 9311 and CSSL29. Data represent mean FPKM values from three biological replicates. The p-value is obtained from the T-test.

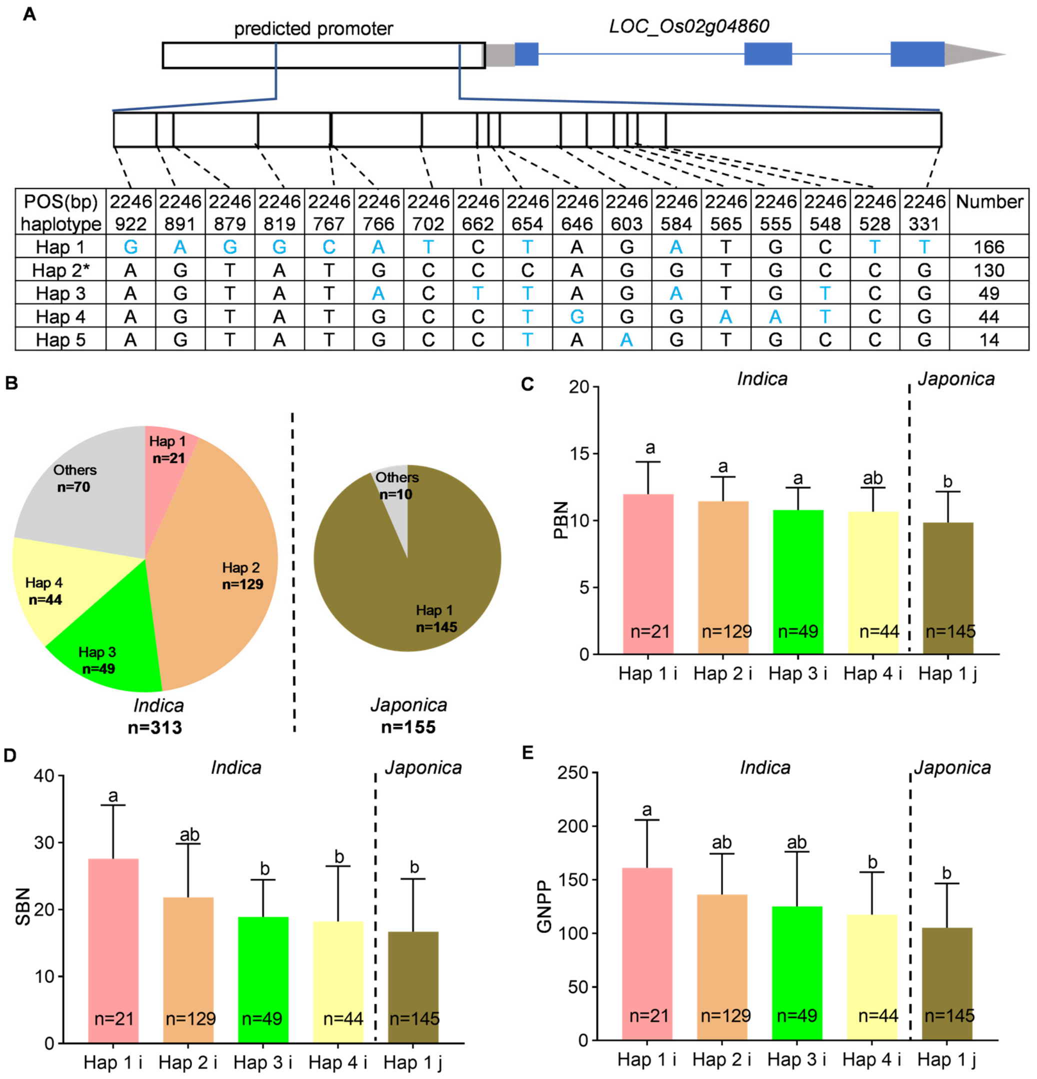

POS(bp) haplotype	2246 922	2246 891	2246 879	2246 819	2246 767	2246 766	2246 702	2246 662	2246 654	2246 646	2246 603	2246 584	2246 565	2246 555	2246 548	2246 528	2246 331	Number
Hap 1	G	A	G	G	C	A	T	C	T	A	G	A	T	G	C	T	T	166
Hap 2*	A	G	T	A	T	G	C	C	C	A	G	G	T	G	C	C	G	130
Hap 3	A	G	T	A	T	A	C	T	T	A	G	A	T	G	T	C	G	49
Hap 4	A	G	T	A	T	G	C	C	T	G	G	G	A	A	T	C	G	44
Hap 5	A	G	T	A	T	G	C	C	T	A	A	G	T	G	C	C	G	14

Figure S4. Haplotype analysis of *LOC_Os02g04860*. (A) Schematic representation of *LOC_Os02g04860* structure and the positions of 17 SNPs used for haplotype analysis. SNPs that differ from the reference sequence are marked with blue letters. (B) The haplotype frequency distribution of *LOC_Os02g04860* in two subpanels, *indica* and *japonica*. (C) Comparison of PBN among accessions with different haplotypes in two subpanels, *indica* and *japonica*. (D) Comparison of SBN among accessions with different haplotypes in two subpanels, *indica* and *japonica*. (E) Comparison of GNPP among accessions with different haplotypes in two subpanels, *indica* and *japonica*. * indicates the haplotype is the same as NPB. Others in the pie-chart mean the haplotypes with a frequency of less than ten. The i and j behind Hap in (C-E) indicate *indica* accessions and *japonica* accessions with relevant haplotypes, respectively. Different lowercase letters indicate significant differences among accessions with different haplotypes based on Duncan's new multiple-range test ($p < 0.05$).

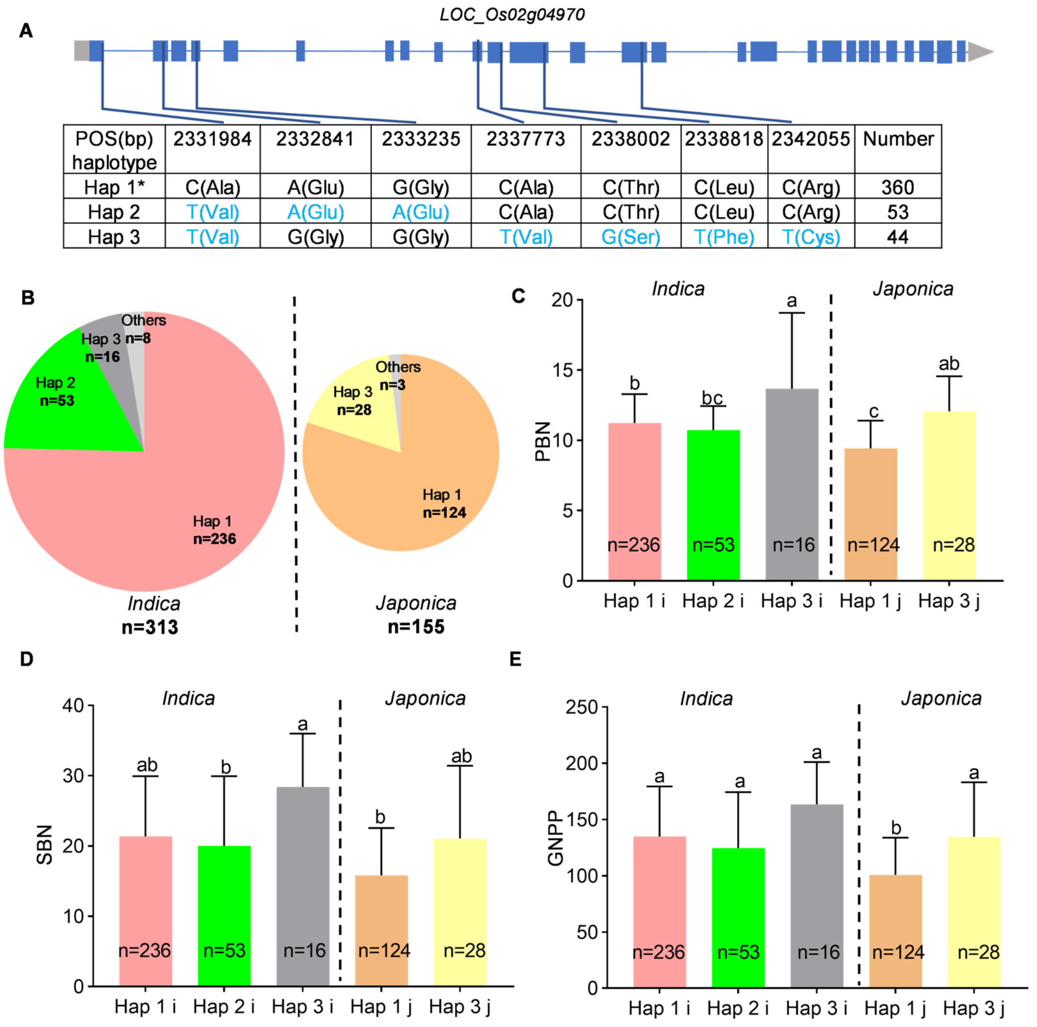

POS(bp) haplotype	2331984	2332841	2333235	2337773	2338002	2338818	2342055	Number
Hap 1*	C(Ala)	A(Glu)	G(Gly)	C(Ala)	C(Thr)	C(Leu)	C(Arg)	360
Hap 2	T(Val)	A(Glu)	A(Glu)	C(Ala)	C(Thr)	C(Leu)	C(Arg)	53
Hap 3	T(Val)	G(Gly)	G(Gly)	T(Val)	G(Ser)	T(Phe)	T(Cys)	44

Figure S5. Haplotype analysis of *LOC_Os02g04970*. (A) Schematic representation of *LOC_Os02g04970* structure and the positions of seven nonsynonymous SNPs used for haplotype analysis. SNPs that differ from the reference sequence are marked with blue letters. (B) The haplotype frequency distribution of *LOC_Os02g04970* in two subpanels, *indica* and *japonica*. (C) Comparison of PBN among accessions with different haplotypes in two subpanels, *indica* and *japonica*. (D) Comparison of SBN among accessions with different haplotypes in two subpanels, *indica* and *japonica*. (E) Comparison of GNPP among accessions with different haplotypes in two subpanels, *indica* and *japonica*. * indicates the haplotype is the same as NPB. Others in the pie-chart mean the haplotypes with a frequency of less than ten. The i and j behind Hap in (C-E) indicate *indica* accessions and *japonica* accessions with relevant haplotypes, respectively. Different lowercase letters indicate significant differences among accessions with different haplotypes based on Duncan's new multiple-range test ($p < 0.05$).

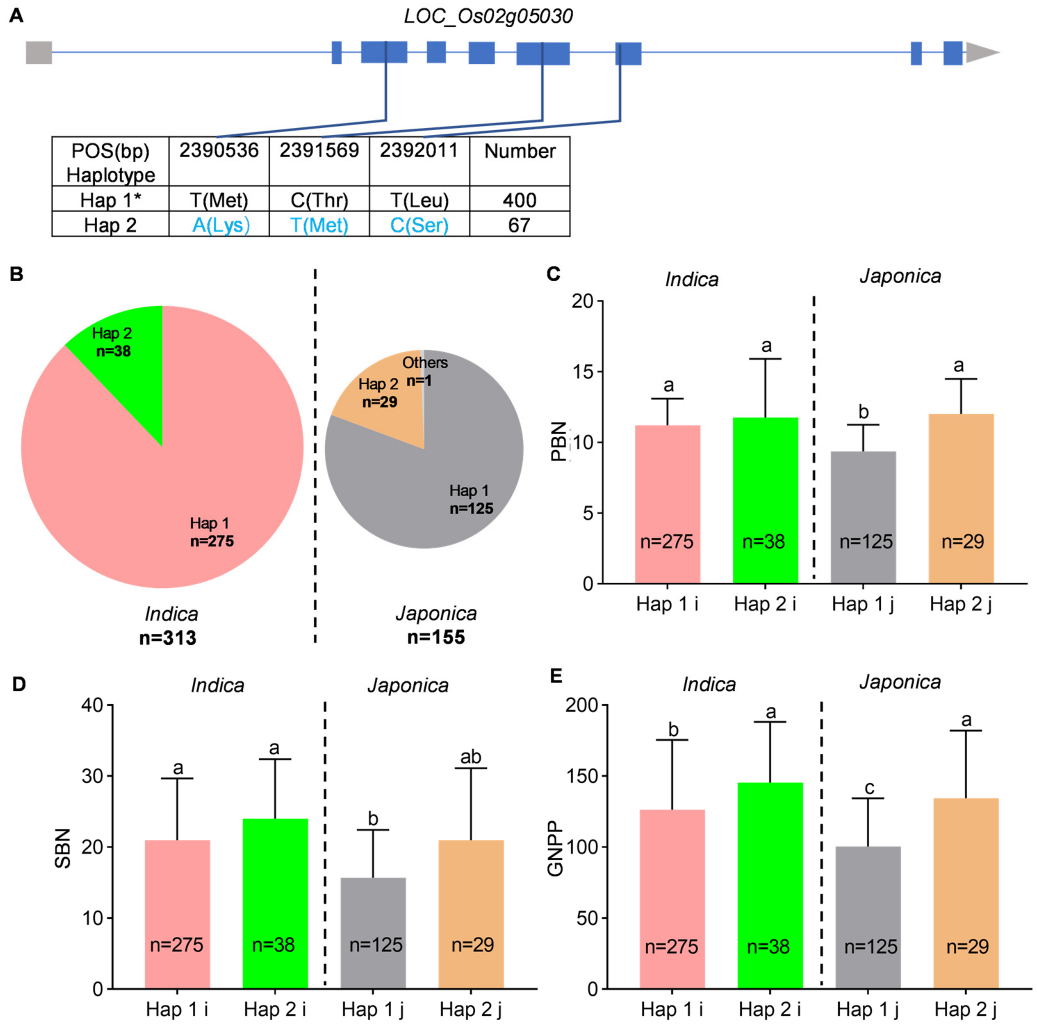

Figure S6. Haplotype analysis of *LOC_Os02g05030*. (A) Schematic representation of *LOC_Os02g05030* structure and the positions of three nonsynonymous SNPs used for haplotype analysis. SNPs that differ from the reference sequence are marked with blue letters. (B) The haplotype frequency distribution of *LOC_Os02g05030* in two subpanels, *indica* and *japonica*. (C) Comparison of PBN among accessions with different haplotypes in two subpanels, *indica* and *japonica*. (D) Comparison of SBN among accessions with different haplotypes in two subpanels, *indica* and *japonica*. (E) Comparison of GNPP among accessions with different haplotypes in two subpanels, *indica* and *japonica*. * indicates the haplotype is the same as NPB. Others in the pie-chart mean the haplotypes with a frequency of less than ten. The i and j behind Hap in (C-E) indicate *indica* accessions and *japonica* accessions with relevant haplotypes, respectively. Different lowercase letters indicate significant differences among accessions with different haplotypes based on Duncan's new multiple-range test ($p < 0.05$).

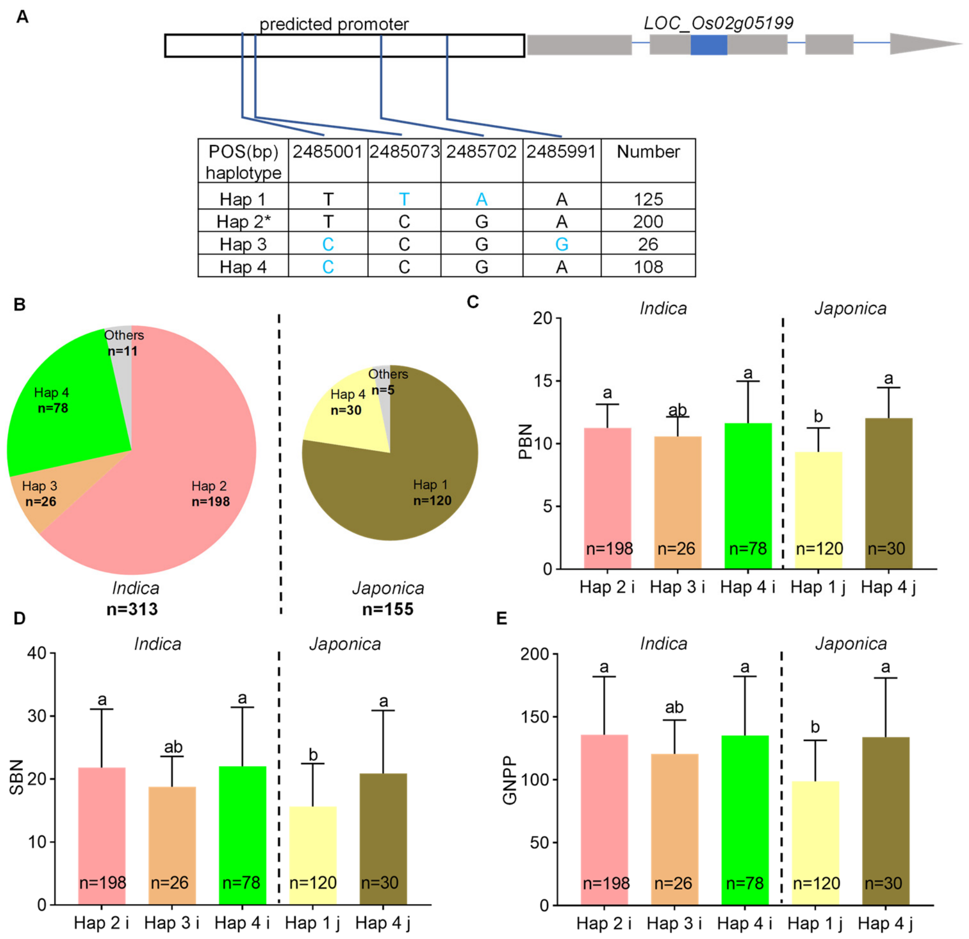

Figure S7. Haplotype analysis of *LOC_Os02g05199*. (A) Schematic representation of *LOC_Os02g05199* structure and the positions of four SNPs used for haplotype analysis. SNPs that differ from the reference sequence are marked with blue letters. (B) The haplotype frequency distribution of *LOC_Os02g05199* in two subpanels, *indica* and *japonica*. (C) Comparison of PBN among accessions with different haplotypes in two subpanels, *indica* and *japonica*. (D) Comparison of SBN among accessions with different haplotypes in two subpanels, *indica* and *japonica*. (E) Comparison of GNPP among accessions with different haplotypes in two subpanels, *indica* and *japonica*. * indicates the haplotype is the same as NPB. Others in the pie-chart mean the haplotypes with a frequency of less than ten. The i and j behind Hap in (C-E) indicate *indica* accessions and *japonica* accessions with relevant haplotypes, respectively. Different lowercase letters indicate significant differences among accessions with different haplotypes based on Duncan's new multiple-range test ($p < 0.05$).

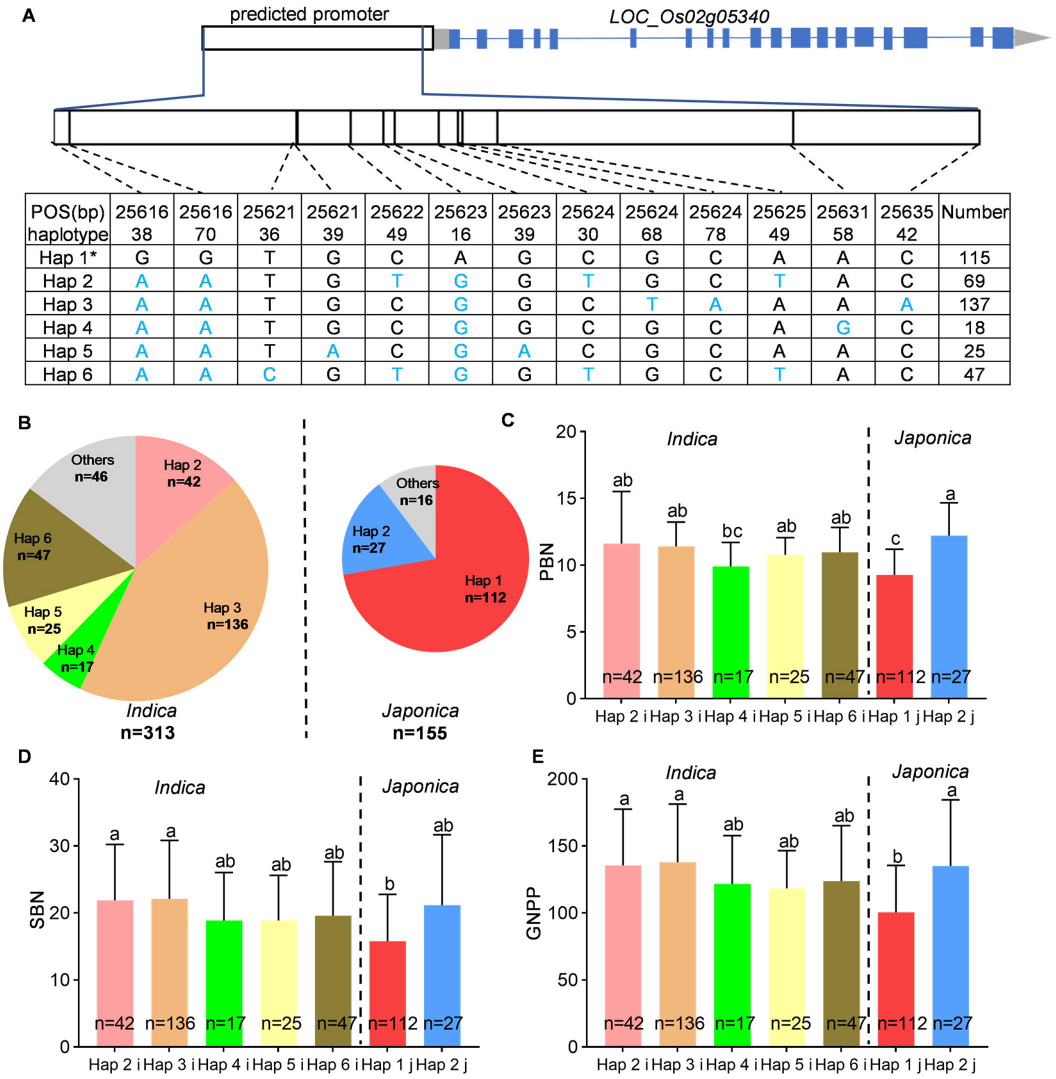

Figure S8. Haplotype analysis of *LOC_Os02g05340*. (A) Schematic representation of *LOC_Os02g05340* structure and the positions of 13 SNPs used for haplotype analysis. SNPs that differ from the reference sequence are marked with blue letters. (B) The haplotype frequency distribution of *LOC_Os02g05340* in two subpanels, *indica* and *japonica*. (C) Comparison of PBN among accessions with different haplotypes in two subpanels, *indica* and *japonica*. (D) Comparison of SBN among accessions with different haplotypes in two subpanels, *indica* and *japonica*. (E) Comparison of GNPP among accessions with different haplotypes in two subpanels, *indica* and *japonica*. * indicates the haplotype is the same as NPB. Others in the pie-chart mean the haplotypes with a frequency of less than ten. The i and j behind Hap in (C-E) indicate *indica* accessions and *japonica* accessions with relevant haplotypes, respectively. Different lowercase letters indicate significant differences among accessions with different haplotypes based on Duncan's new multiple-range test ($p < 0.05$).

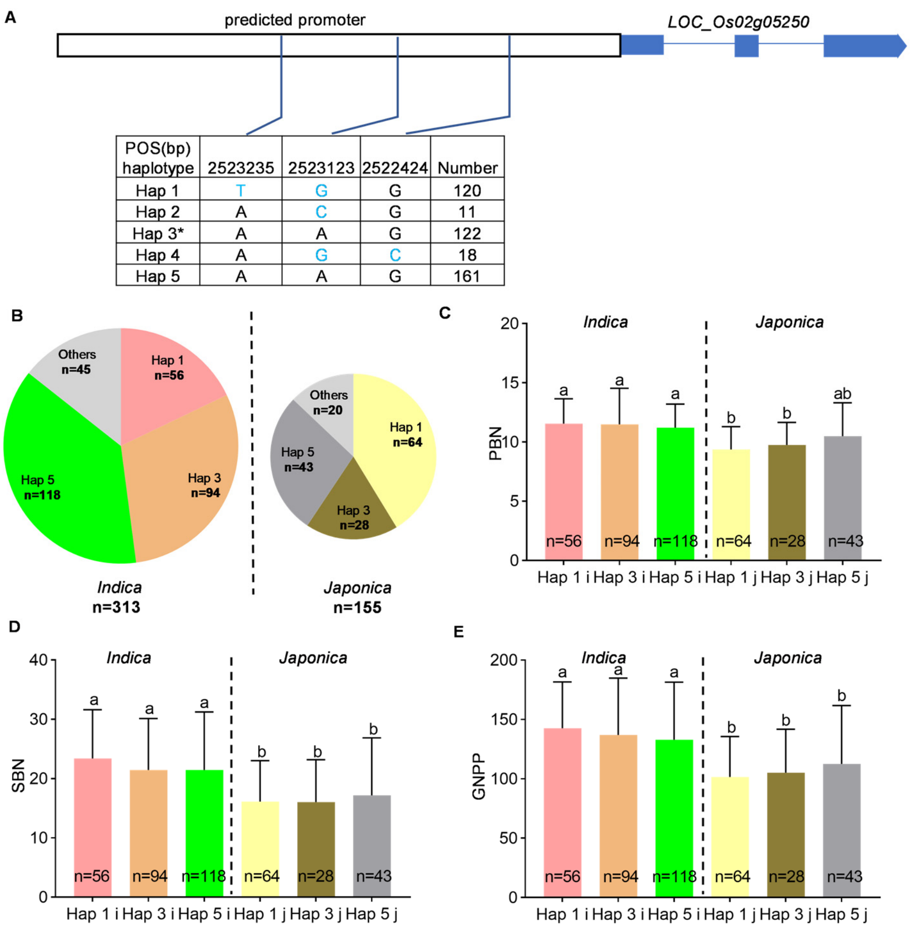

Figure S9. Haplotype analysis of *LOC_Os02g05250*. (A) Schematic representation of *LOC_Os02g05250* structure and the positions of three SNPs used for haplotype analysis. SNPs that differ from the reference sequence are marked with blue letters. (B) The haplotype frequency distribution of *LOC_Os02g05250* in two subpanels, *indica* and *japonica*. (C) Comparison of PBN among accessions with different haplotypes in two subpanels, *indica* and *japonica*. (D) Comparison of SBN among accessions with different haplotypes in two subpanels, *indica* and *japonica*. (E) Comparison of GNPP among accessions with different haplotypes in two subpanels, *indica* and *japonica*. * indicates the haplotype is the same as NPB. Others in the pie-chart mean the haplotypes with a frequency of less than ten. The i and j behind Hap in (C-E) indicate *indica* accessions and *japonica* accessions with relevant haplotypes, respectively. Different lowercase letters indicate significant differences among accessions with different haplotypes based on Duncan's new multiple-range test ($p < 0.05$).
